# Extra-legal abortion and post-abortion care knowledge, attitudes, and practices among obstetrician-gynecologist clinicians and medical residents in San José, Costa Rica: a qualitative study

**DOI:** 10.1186/s12905-023-02639-y

**Published:** 2023-09-21

**Authors:** Blake Erhardt-Ohren, Ellyn Pier, Daniel Arroyo, Whitney Cole, McKaylah Hilliard, Adriana Otero-Gonzalez, Oscar Hidalgo-Mora, Sebastian Ospina-Henao, Roger Rochat, Anna Newton-Levinson

**Affiliations:** 1 School of Public Health, University of California, Berkeley, USA; 2 Rollins School of Public Health, Emory University, Atlanta, USA; 3 Faculty of Medicine, Universidad de Ciencias Médicas, San José, Costa Rica; 4 Goizueta School of Business, Emory University, Atlanta, USA

**Keywords:** Costa Rica, Abortion, Termination, Miscarriage, Obstetrician, Gynecologist, Resident

## Abstract

**Background:**

Induced abortion in Costa Rica is illegal in all cases except to save the life of the pregnant person. Despite severe restrictions to legal abortion, individuals in Costa Rica still induce abortions outside of the formal healthcare system. These individuals and those with spontaneous abortions, also known as miscarriages, occasionally need medical care for complications. In Costa Rica, an estimated 41% of unintended pregnancies end in abortion, yet there is very little published literature exploring the perspectives of healthcare providers on abortion in Costa Rica.

**Methods:**

We interviewed ten obstetrician-gynecologist clinicians and five obstetrician-gynecologist medical residents in San José, Costa Rica about their beliefs and practices related to extra-legal abortion and post-abortion care (PAC) using a Spanish language in-depth semi-structured interview guide. After transcription and translation into English, analysis team pairs used a combination of deductive and inductive coding to identify themes and sub-themes within the data.

**Results:**

Obstetrician-gynecologist clinicians and medical residents were aware of the presence of extra-legal abortion, and particularly, medication abortion, in their communities, but less familiar with dosing for induction. They expressed the desire to provide non-judgmental care and support their patients through extra-legal abortion and PAC journeys. Study participants were most familiar with providing care to individuals with spontaneous abortions. When discussing PAC, they often spoke about a policy of reporting individuals who seek PAC following an extra-legal abortion, without commenting on whether or not they followed the guidance.

**Conclusions:**

This study contributes to a gap in research about the knowledge, attitudes, and practices of Costa Rican obstetrician-gynecologist clinicians and medical residents around extra-legal abortion and PAC. The results reveal an opportunity to train these healthcare providers as harm reduction experts, who are able to accurately counsel individuals who are seeking abortion services outside of the healthcare system, and to provide training to improve care for individuals needing PAC.

## Introduction

 Since 1970, Costa Rica’s penal code has criminalized abortion except “to save the life or health of the woman“ [1]. Under this policy, both an individual receiving an extra-legal abortion service, an abortion that is managed outside of the country’s legal system, and the individual who provided the service can be prosecuted [1]. In late 2019, the Norma Técnica protocol, or *Technical Standard*, was introduced by the Costa Rican Ministry of Health to provide further guidance to clinicians on providing life-saving abortions and outlined restrictions on other abortion services [2]. However, research worldwide has shown that restricting access to legally induced abortion does not prevent individuals from seeking out abortion services [3]. In Costa Rica, the Guttmacher Institute estimates that between 2015 and 2019, 41% of unintended pregnancies ended in induced abortion [4]. There exist no estimates for the number of induced abortions that are performed extra-legally, the number of individuals experiencing complications from unsafe abortion, or the number of individuals with abortion complications that require medical treatment in Costa Rica [5].

Globally, 7.9% of maternal deaths are estimated to be attributable to unsafe abortion [6], demonstrating the life-saving importance of comprehensive abortion services, which includes both access to safe abortion and post-abortion care (PAC). Unsafe abortion refers to procedures that are either not provided by a trained professional, not carried out using a method appropriate to pregnancy duration, or both [7]. Not all extra-legal abortions are unsafe abortions, and the World Health Organization has published guidance on the use of self-managed medication abortion, which is both effective and safe [8–12]. PAC involves counseling to respond to the patient’s needs, treatment for incomplete abortion and complications arising from abortion, and the provision of family planning and other reproductive health services [13]. Reasons for the high proportion of deaths due to unsafe abortion are manifold, as individuals may not be able to access safe, legal abortions, may not be able to access PAC for complications following a spontaneous or induced abortion, or, even with access to medical institutions, may encounter healthcare professionals who decline to provide care [14–21]. Despite these and other barriers, some evidence has shown that healthcare professionals may be supportive of providing lifesaving PAC, though they are constrained by the health institutions in which they work [22].

Few published research articles have explored provider-based barriers to comprehensive abortion care in Latin American countries where abortion is highly restricted [23–27]; of the studies we did find, three are policy or historical analyses [23–25], one is a survey of clients [27], and only one directly engages with healthcare providers [26]. We found one published article from Brazil that included medical residents and not solely clinicians, though it only explored knowledge about medication abortion and not abortion provision practices [28]. We were unable to find any published literature investigating clinician perceptions and practices around extra-legal abortion or PAC in Costa Rica despite its restrictive policy environment and the estimated high frequency of extra-legal and/or unsafe abortion. We sought to fill this gap in the research by conducting semi-structured in-depth interviews with obstetrician-gynecologist (OB/GYN) clinicians and medical residents in San José, Costa Rica.

## Methods

This study was co-designed by researchers at Emory University, the Universidad de Ciencias Médicas (UCIMED), the largest private medical university in Costa Rica, and the University of California, Berkeley. This manuscript focuses on data from interviews with healthcare professionals and their knowledge, attitudes, and practices related to extra-legal abortion and PAC. Throughout the article, we specifically refer to “extra-legal” abortion to clarify that not all induced abortions performed outside of formal healthcare systems are unsafe.

Between September 2021 and March 2022, OB/GYN clinicians and medical residents were recruited via email, WhatsApp, and flyers disseminated by UCIMED through internal list servs. These flyers were disseminated to individuals at both public and private facilities. Due to the structure of the Costa Rican healthcare system and our interest in recruiting the individuals most likely to perform abortions, we specifically sought clinicians and medical residents in tertiary facilities – those providing specialized care. However, we did not ask participants to disclose their place of work, and therefore, cannot report on the number of public and private facilities included in the results, only the number of clinicians that worked at public and private facilities. After a few initial interviews, snowball sampling was used to recruit additional participants. Clinicians were considered eligible to participate if they were currently practicing as an OB/GYN in Costa Rica and medical residents were considered eligible to participate if they were within the OB/GYN specialty in Costa Rica. There were no exclusion criteria related to age, race, or gender.

Semi-structured in-depth interviews were employed due to the sensitive nature of the study topic. Interview guides were developed to explore healthcare professional knowledge and interpretation of current abortion laws, perspectives on the provision of comprehensive abortion care, and hopes and beliefs about the future of abortion service provision in Costa Rica. These interview guides included further iterative probes in order to more fully explore themes that arose. All interview guides and informed consent materials were first authored in English and then translated into Spanish. Once in Spanish, the materials were translated back into English as a validation measure. As required by Costa Rican law, all researchers collecting data completed an ethics course. The study protocol and all materials were approved by the Emory University Institutional Review Board (#STUDY00002394) and the UCIMED Ethics Committee (#586-06-2021) in August 2021.

Participants scheduled interviews using an online calendar tool. Interviews were conducted in Spanish using virtual Zoom conferencing software with audio only. Before interviews, researchers at UCIMED collected both verbal and written informed consent from all study participants. In all interviews, one researcher conducted the interview while a second researcher took detailed notes. Interview recordings were transcribed in Spanish and translated into English by a professional translation company specializing in the Costa Rican Spanish dialect. Study team researchers fluent in both Spanish and English verified each translation, as did native Spanish-speaking study team members. De-identified transcripts were analyzed in MaxQDA using content analysis. The team developed a codebook of deductive and inductive codes. To ensure consistency, at least two individuals analyzed each transcript. Codes were refined through team discussion and inter-coder agreement assessments. We concluded recruitment once we achieved code and meaning saturation.

## Results

Ten OB/GYN clinicians and five OB/GYN medical residents participated in the study. Eight participants identified as male and seven identified as female. The average age of clinicians was 45.6 years, and the average age of medical residents was 30.0 years. Almost half of the participants were Catholic (*n* = 7) and five expressed some kind of spirituality. The average length of practice for clinicians was 20.0 years and the average length of practice for residents was 2.2 years. All participants worked in the public sector, with eight also working part-time in the private sector. The majority (*n* = 12) of participants worked in tertiary facilities and fourteen identified the facilities in which they worked as urban. Table 1 displays further demographic details of study participants and characteristics of the facility in which they worked.

**Table 1:** Demographic characteristics of study participants

	Clinicians (*n* = 10)	Medical residents (*n* = 5)
Gender		
* Male*	4	4
* Female*	6	1
* Other*	0	0
Age		
* 20–29 years*	0	2
* 30–39 years*	3	3
* 40–49 years*	4	0
* 50 + years*	3	0
Religion		
* Catholic*	4	3
* Non-Catholic Christian*	2	0
* Agnostic*	2	1
* No religion*	2	1
Years in practice		
* 0–9*	2	5
* 10–19*	4	0
* 20–29*	1	0
* 30+*	3	0
Health facility location		
* Urban*	9	5
* Rural*	1	0
Health facility sector		
* Public*	2	5
* Private*	0	0
* Both*	8	0
Health facility sector		
* Primary*	2	0
* Secondary*	1	0
* Tertiary*	7	5

### Knowledge of extra-legal abortion and PAC

All clinicians and medical residents expressed knowledge of at least one way that individuals induce abortions themselves. Participants identified medication as the most common method to induce abortion outside of the formal health system; some participants referred only to this generically as “medicine,” whereas others specifically called out the use of prostaglandins for induced termination of pregnancy. For example, one clinician stated:

“Well, here there has always been a clandestine market of prostaglandins, of misoprostol, so patients buy them and take them, ingest them, apply them intravaginally and thus begin their process of terminating their pregnancy.” – Clinician, male, 61 years old.

While participants were aware of the use of medication to induce abortion and the process by which it worked, study participants were less knowledgeable about the correct dosage, with one clinician saying:

“… I also have no experience prescribing dosage and prescribing treatments of induced abortions, so I do not feel comfortable giving advice that I have not done and that I do not feel comfortable doing…” – Clinician, male, 31 years old.

Instead, study participants discussed their familiarity with providing care to individuals who spontaneously miscarried, noting that “there is a guide for abortions, but the abortion guide that exists… are abortion guides for spontaneous abortion” (Clinician, male, 58 years old).

Regarding medication abortion, most study participants discussed their awareness of the use of medication abortion, procured extra-legally, among their patients. They often noted their inability to distinguish between induced and spontaneous abortions:

“… I’ve probably given care to more than one [induced abortion client], but without them telling me it was induced. On the other hand, they just arrive at the institution with the abortion happening, and we don’t have a way to know if it was induced or not. So, well, we put them in, too, the induced ones and the spontaneous ones together.” – Clinician, female, 42 years old.

A few study participants were more specific, stating that if an individual had used abortion medication to induce a termination of pregnancy that was accomplished, “… through pills… they have already been dissolved when [patients] come in for a consult” (Clinician, male, 40 years old). The same clinician noted that individuals are unlikely to disclose that they had induced an extra-legal abortion, unless speaking out of fear for their health.

Study participants also discussed other ways that individuals induce termination of pregnancy or seek abortion outside of the Costa Rican healthcare system. Methods discussed included travel to other countries where abortion medication could be purchased over the counter, or where safe abortion services could be provided legally, using Google to locate abortion services, or finding a private clinician in Costa Rica to provide services clandestinely. Only one clinician suggested that individuals with an unwanted pregnancy would insert a foreign object to induce an abortion, while another stated that she did not believe foreign objects were used to induce abortion in Costa Rica anymore.

### Attitudes towards extra-legal abortion and PAC

More than half of the study participants spoke about the importance of non-judgement when providing PAC for both individuals with spontaneous abortions and individuals that had obtained extra-legal abortions. One clinician, who, unlike others, believed that he could distinguish between induced and spontaneous abortions, stated:

“Yes, many times it is known. One touches and pulls misoprostol pills from [the] back of the vagina. And you know that happened, but you don’t judge.” – Clinician, male, 58 years old.

These same participants emphasized the importance of providing support to the patients under their care. They defined this care as providing not only healthcare but emotional and educational support. One medical resident spoke at length about his beliefs about what happens when individuals seek out extra-legal abortion services, and the role that a clinician should play versus what actually happens:

“… It seems to me that there should be a personal connection… So, my feeling is that it’s due to the fact that it’s an illegal situation, so to speak, [there is] … a lot of abandonment of the patient and perhaps a very lonely experience for the woman.” – Medical resident, male, 33 years old.

Ultimately, many study participants spoke about the positive feelings they have providing care to individuals in need. They clarified that their job was to provide care in line with what the patient needed:

“I feel good from the point of view that I … tend to be a professional who gives you the appropriate support. And I’m clear that I give it even when I know it was an induced abortion… It is something that happened, that she decided and that’s it. And, as I tell you, it doesn’t change my care. I see a miscarriage as exactly the same as an induced abortion. So, I feel like I’m at least contributing from that side as well as respecting their decision. I think I feel, let’s just say good.” – Clinician, male, 31 years old.

A medical resident summarized the experience of many other study participants succinctly emphasizing the importance of empathy:

“I think this is more about empathy with the patients. Knowing how to understand them, to put yourself in their shoes, seems to me to be important. So I do consider that I have the ability or the facility to be empathetic with patients.” – Medical resident, female, 28 years old.

Despite the illegality of most induced abortions, many study participants did not express bias towards individuals who needed treatment for complications from unsafe abortions. Instead, they highlighted their role as a sympathetic healthcare provider to all individuals in need.

### Practices around extra-legal abortion and PAC

When asked about providing induced abortion services, study participants almost always first stated that induced abortions were not provided at their place of work. No participants disclosed direct experience with providing extra-legal abortions or those permitted within the Norma Técnica. Most participants did describe receiving training to care for pregnant individuals experiencing incomplete spontaneous abortion. As one clinician explained:

“… [In medical residency] we learned very little about abortion techniques. We just learned in the specialty, but for retained abortions, little ones who died inside the womb. Things like that.” – Clinician, male, 58 years old.

After discussing their learning about how to provide PAC for retained products of conception, study participants often spoke about their actual practice. The same study participants reiterated that the techniques they learned in medical school and residency were targeted towards evacuating fetal remains after a spontaneous abortion. They were not trained to induce abortions, but only provided services to patients who were carrying a deceased fetus. Participants did not discuss the use of medication to remove fetal remains, but instead spoke about expectant management. As one participant explained:

“Okay, if you have an abortion it means the fetus is dead. That is, if the fetus has already died, then there are several alternatives for management. One, which is expectant management, which is waiting until the body induces the termination of pregnancy, either with contractions, bleeding and all that. Two, here we don’t have prostaglandins. Well, we have Dinoprostone [prostaglandin E2]. We don’t have misoprostol for use.” – Clinician, male, 61 years old.

With regards to suspected extra-legal abortions, clinicians and medical residents spoke mostly about policies and procedures that they were expected to follow and not their own personal practices:

“If she arrives at the hospital and has the misoprostol pill in her vagina, you have to call the OIJ, the Judicial Investigation Agency; and the Judicial Investigation Agency begins the process, because she is being tried for homicide… then, the police arrive and if the girl is not unstable or if she does not require emergency medical attention, they even take her away.” – Clinician, female, 48 years old.

Although study participants stated that there was sometimes difficulty determining who had induced an abortion versus suffered a spontaneous abortion, only one study participant specifically stated that clinicians would not notify authorities if they knew they were caring for a patient who had sought extra-legal abortion services, saying:

“Nothing happens, they are not interrogated, the police are not going to be called to do something to them. They are given care, the uterus is cleaned and they are offered a method of postabortion planning, psychological counseling if necessary and that’s it, done. Nothing happens.” – Clinician, male, 58 years old.

No study participants discussed their personal opinion about their place of work’s policies or procedures, but rather, detailed the steps that they were expected to take when confronted with potential illegal activity without disclosing what they would do themselves.

## Discussion

Our study contributes to a small but growing literature documenting healthcare provider attitudes and practices around PAC and extra-legal abortion in Latin American countries. We found that although OB/GYN clinicians and medical residents are aware of the use of extra-legal abortion in their communities and some abortion induction methods, they were less familiar with how those methods were used. This aligns with research conducted in other restrictive countries such as Brazil [28] and Burkina Faso [18]. Instead, study participants were more comfortable speaking about PAC after spontaneous abortions, though they noted that they were often unable to distinguish the cause of the complications requiring PAC.

Most participants spoke about wanting to provide better services to patients and to help them to lead healthy lives and prevent adverse pregnancy-related outcomes. There appear to be opportunities to explore harm reduction in this cadre of providers since they desire to accompany patients and ensure that they are healthy. These clinicians and medical residents expressed positive feelings about treating individuals with complications from abortion, no matter the cause, and emphasized the desire to be non-judgmental and to support their patients throughout the process. This is in line with the development of PAC to address unsafely induced abortion and supports the notion of expanding access to PAC training in the country [13]. Regarding practice, study participants spoke about policies and procedures at their respective medical institutions, and that training only covered the removal of fetal remains. They also spoke about the expectation that they would comply with their institutions’ policies and procedures, including reporting patients suspected of inducing abortion extra-legally. Except for one clinician, study participants did not specifically state whether or not they followed these procedures. The results of our study align with another, conducted in Tunisia, that showed that though healthcare providers may not have negative feelings toward providing services, their actions may be limited by their health institution [22]. We did not find the same religious objection to provision of abortion care that was found in studies conducted in Nigeria and Uganda [17, 29].

Our study results lead us to believe that there may be opportunities for Costa Rican OB/GYN healthcare providers to be trained in harm reduction for unsafe abortion. This model, conceptualized in Uruguay in 2002, prepared clinicians to provide comprehensive and factually correct abortion information to potential abortion seekers [30]. Harm reduction led to improvements in reproductive health service delivery, decreases in maternal morbidity and mortality, and increased visibility and support of sociopolitical movements to support women’s rights [30]; implementers in other countries, such as Peru and Tanzania, have conducted studies on the model’s implementation and found support for its use [31, 32]. Our findings support an introduction of the model in Costa Rica, where it may similarly improve reproductive health outcomes.

### Limitations and strengths

This study is limited by the lack of diversity among participants. Participants in this study almost exclusively worked in urban public health facilities. The results of the study should be interpreted within that context and may not be applicable to healthcare providers in other types of medical establishments in Costa Rica. In addition, the use of conferencing software may have made participants reluctant to share more information. However, some research has shown that virtual interviews may actually result in less participant inhibition and more comfort with disclosing sensitive information in interviews [33–35]. This study benefited from the robust collaboration between Emory University, UCIMED, and the University of California, Berkeley researchers and the seamlessness of data collection, analysis, and interpretation.

## Conclusions

This qualitative exploratory study begins to fill a gap in research related to Costa Rican healthcare provider knowledge, attitudes, and practices around extra-legal abortion and PAC and identifies several areas to further address reproductive health needs and provider training. These providers expressed their desire to give non-judgmental care and support individuals through their PAC and sometimes, extra-legal abortion experience. Our study findings point to an opportunity to train these providers to become harm reduction experts, who can share important information to reduce the risk of morbidity and mortality for individuals seeking out abortion outside of the formal healthcare system. There are further opportunities for providers to receive critical training in both medical school and residency to improve their ability to adequately care for individuals that require PAC after an extra-legal and/or unsafe abortion. We look forward to future research that explores these possibilities.

## Data Availability

The datasets generated and analyzed during the current study are not publicly available due to them containing information that could compromise research participant privacy, but are available from the corresponding author on reasonable request.

## Abbreviations

OB/GYN: Obstetrician-gynecologist
PAC: Post-abortion care
UCIMED: Universidad de Ciencias Médicas
